# Emerging risk factors for nonalcoholic fatty liver disease associated hepatocellular carcinoma

**DOI:** 10.20517/2394-5079.2020.16

**Published:** 2020-06-18

**Authors:** Jihane N. Benhammou, Jonathan Lin, Shehnaz K. Hussain, Mohamed El-Kabany

**Affiliations:** 1 Pfleger Liver Institute, University of California Los Angeles, Los Angeles, CA 90095, USA; 2 Vatche and Tamar Manoukian Division of Digestive Diseases, David Geffen School of Medicine at UCLA, Los Angeles, CA 90095, USA; 3 Department of Epidemiology, Fielding School of Public Health, University of California, CA 90095, USA; 4 Department of Medicine, Samuel Oschin Comprehensive Cancer Institute, Cedars-Sinai Medical Center, Los Angeles, CA 90048, USA

**Keywords:** Nonalcoholic fatty liver disease, nonalcoholic steatohepatitis, hepatocellular carcinoma, statins, metabolic syndrome

## Abstract

Worldwide, nonalcoholic fatty liver disease (NAFLD) has reached epidemic proportions and in parallel, hepatocellular carcinoma (HCC) has become one of the fastest growing cancers. Epidemiological studies have not only shed light on the prevalence and incidence of the disease but have also unmasked important environmental risk factors, including the role of diabetes and dyslipidemia in disease pathogenesis. Genetic association studies have identified single nucleotide polymorphisms implicated in NAFLD-HCC, many of which are part of lipid metabolism pathways. Through these clinical studies and subsequently, translational and basic research, the role of statins as a chemoprotective agent has also emerged with ongoing clinical trials assessing their utility in HCC prevention and treatment. In this review, we summarize the recent epidemiological studies describing the burden of NAFLD-HCC in different patient populations and countries. We discuss the genetic and environmental risk factors for NAFLD-HCC and highlight the chemoprotective role of statins and aspirin. We also summarize what is known about NAFLD-HCC in the cirrhosis and non-cirrhosis populations and briefly address the role of surveillance in NAFLD-HCC patients.

## INTRODUCTION

The metabolic syndrome (MetS), defined by the clustering of biochemical and clinical features, which includes type 2 diabetes (T2D), hypertension, dyslipidemia and obesity, has reached epidemic proportions^[1]^.

Non-alcoholic fatty liver disease (NAFLD), the liver manifestation of MetS, has increased in parallel and is now the most common cause of liver disease in the United States^[2]^. Although the true prevalence of NAFLD remains unknown given the lack of validated and/or recommended screening practices, it is estimated that the disease affects about a quarter of the world’s population, depending on geographical differences^[3]^. NAFLD can progress to nonalcoholic steatohepatitis (NASH)^[3]^ (characterized by ≥ 5% of hepatic steatosis with lobular inflammation and hepatocyte ballooning^[4]^), cirrhosis and hepatocellular carcinoma (HCC)^[5,6]^. Given the estimated increase in NAFLD, NASH and NAFLD-associated HCC^[7]^ and the anticipated burden on health care costs^[8–10]^, several studies have focused on understanding the clinical and biological drivers of NAFLD-associated HCC and its potential treatment options.

Understanding this disease process is especially relevant since NAFLD-associated HCC can occur in a noncirrhotic background^[11–13]^. This poses a clinical dilemma given the lack of screening guidelines for this sub-group of patients, thus prompting the need for further understanding of the natural history of NAFLD-HCC and identifying at-risk populations who would benefit from screening. More recently, studies have also identified the protective effects of statins and aspirin on fibrosis progression and HCC^[14–17]^, providing an avenue for further research in this group of patients who are also at high risk for cardiovascular disease.

A full review and discussion of the pathophysiology of NAFLD and NASH is beyond the scope of this report and has been summarized by Anstee *et al.*^[18]^ In this review, we explore what is known about the genetic (non-modifiable) and environmental (modifiable) risk factors of NAFLD-associated HCC, examine the role of statins and aspirin and what microbiome research has to offer in the field of NAFLD-related HCC.

## BURDEN OF NAFLD-ASSOCIATED HCC

HCC is a lethal cancer with a rising incidence over the last 30 years^[19]^. Its incidence is increasing most rapidly of any cancer, with an age-adjusted annual increase of 3.8% and 2.8% in men and women in the U.S., respectively^[20]^. The rising HCC burden has largely been attributed to the rise in obesity and diabetes^[21]^. As follows, several epidemiological studies have specifically examined the incidence and risk of NAFLD-associated HCC [Table 1]. The results have been varied however, due to differences between the studies in patient population, time-period, and NAFLD and/or NASH ascertainment. For example, in a large Veterans Affairs (VA) Health System study between 2003-2011, the incidence of HCC in a NAFLD cohort was 0.21 per 1000 person-years^[22]^. A separate study within the VA further demonstrated that of the 1500 HCC cases identified from 2005-2011, NAFLD was the underlying risk factor in 8% of all cases with an annual proportion of NAFLD-related HCC ranging from 7.5%-12.0%^[23]^. Ioannou *et al.*^[24]^ also reported that the incidence of NAFLD-associated HCC was 1.56% within the VA from 2012-2018 over a 3.7 years follow up period. In non-VA populations, the incidence rate for NAFLD-associated HCC and NASH-associated HCC were 0.44 and 5.29 per 1000 person-years, respectively^[3]^.

Changes in liver transplantation (LT) indications are also reflective of the increasing rates of NAFLD. For instance, Younossi *et al.*^[25]^ demonstrated that of 158,347 LT candidates from 2002-2016, the prevalence of NAFLD-associated HCC increased by 11.8 fold, which was higher than hepatitis B, hepatitis C and alcoholic liver disease. Other studies have also corroborated that NAFLD and NAFLD-associated HCC are the most rapidly growing indications for LT^[26,27]^.

Other countries in Europe and Asia have made similar observations to these U.S.-based studies^[28,29]^. In a European Electronic Health Record study of patients seen in primary care in Spain, Italy, the Netherlands and the United Kingdom, the incidence of NAFLD-associated HCC diagnosis was 0.3 per 1000 person-years over a median follow up of 3.3 years^[30]^. A study of the European Liver Transplant Registry database from 2002-2016 also showed that among the 68,950 transplant patients, 8.4% were for NASH in 2016 (compared to 1.3% in 2002), 39% of whom had HCC^[31]^. In Asian countries, where hepatitis B has been the main driver of liver disease and HCC, the prevalence of NAFLD is also about 25% depending on the country studied, ranging from 6.2% in South China to 51% in Indonesia^[3,32,33]^. NAFLD-associated HCC has also increased in countries such as South Korea where its prevalence rose from 3.8% (2001-2005) to 12.2% (2006-2010)^[34]^.

Although a rise in sedentary life styles leading to increase in MetS and diabetes are thought to be the culprits in NAFLD-associated HCC, Asian patients are more likely to have “lean” or “non-obese” NAFLD, potentially representing a pathophysiolocally different group of patients from those seen in Western countries. Few studies have addressed this although a recent European study assessing the outcomes of biopsy-proven lean NAFLD (BMI < 25 kg/cm^2^; *n* = 123) patients compared to healthy controls, overweight NAFLD (BMI 25–30 kg/cm^2^; *n* = 335) and obese NAFLD patients (BMI > 30 kg/cm^2^; *n* = 188) demonstrated that lean NAFLD patients had a tendency towards more liver-related complications including cirrhosis, decompensated cirrhosis and HCC over a follow-up period of 19.9 years^[35]^.

In summary, the burden of NAFLD-HCC is on the rise with the prevalence of NAFLD, depending on geographical and ethnic differences, affecting about a quarter of the world’s population. Although NASH is thought to be a more severe form of NAFLD that more commonly progresses to chronic liver disease and HCC, the true prevalence of NAFLD (6.2%-51%) and NASH (10%-20%), based on large epidemiological data, remains unclear given that no specific biomarkers exist to differentiate the two other than a liver biopsy, which is scarcely performed, yet the gold standard for diagnosis.

## GENETIC RISK FACTORS FOR NAFLD-HCC

There is a large body of literature linking genetic polymorphisms to the development of NAFLD and NASH but few have addressed the genetic contributions to NAFLD-associated HCC [Table 2]. Early studies in the field of NAFLD identified ethnic differences in disease prevalence whereby Hispanics were the most commonly affected group followed by Caucasians and African Americans^[36–38]^, suggesting a genetic predisposition to NAFLD. Consistent with these findings, twin studies and phenotypic clustering of fatty liver were also more commonly seen in patients in the same family, suggesting heritability of the disease, which has been reported to range from 38%-50%, depending on the modality used for phenotyping of NAFLD (biopsy, MRI or abdominal ultrasound)^[39,40]^. In recent years, large genome-wide studies have followed and revolutionized what is known about NAFLD and NASH, and possibly the risk for HCC development.

Romeo *et al.*^[41]^ conducted the first genome-wide association study in the Dallas Heart Study using proton magnetic resonance spectroscopy to quantitate hepatic steatosis as the phenotype. Patatin-like phospholipase domain 3, PNPLA3 (rs738409), was the single variant strongly associated with hepatic steatosis. Although the mechanism by which PNPLA3 leads to hepatic steatosis accumulation remains unknown, it was shown to play a role in hepatocellular lipid droplet remodeling and very low-density lipoprotein secretion^[42,43]^. Interestingly, subsequent association studies of PNPLA3 also demonstrated that the variant was associated with histological severity of the disease, thus suggesting that it may influence HCC development, where GG sequence carriers had more necroinflammation and fibrosis compared to CC carriers^[44]^. Indeed, Liu *et al.*^[45]^ demonstrated that in a Northern European Caucasian cohort of patients with primary HCC attributed to NAFLD, carriage of the PNPLA3 rs738409 polymorphism was associated with NAFLD fibrosis and HCC, where GG carriers had a 5-fold increase in HCC (95%CI: 1.47-17.29) compared to CC carriers.

In subsequent studies, the transmembrane 6 superfamily member 2, TM6SF2 (rs58542926), followed suit and was identified in an exome-wide association study of fatty liver and serum aminotransferases^[46]^. Although the association of TM6SF2 with NAFLD is well established, its association with HCC development is disputed^[45,47]^. More recently, the MBOAT7 (Membrane Bound O-Acetyltransferase Domain Containing 7) variant rs641738 has also been associated with NAFLD and its histological severity^[48,49]^. An Italian study of 132 NAFLD-associated HCC cases also linked the MBOAT4 variant to non-cirrhosis NAFLD HCC^[50]^. In another European study, Pelusi *et al.*^[51]^ identified rare variants of candidate genes (*SMAD4, SQSTM1, TEL, RB1, TSC1*), including APOB (Apolipoprotein B) which is involved in very low-density lipoprotein secretion and therefore export of lipids using whole exome sequencing methods. Noteworthy of these findings is the common thread of lipid metabolism genes being identified in genetic NAFLD and NAFLD-related HCC association studies. Although intrahepatic steatosis alone was thought to be benign and the hallmark of NAFLD, lipid dysregulation may play an important role in promoting carcinogenesis independently of NAFLD disease pathogenesis.

In addition to being at risk for NAFLD and NASH, Hispanic patients have been shown to be at risk of NAFLD-associated HCC^[22,52,53]^, which has been attributed largely to an increase in the incidence of cirrhosis^[22]^. Similar to what has been reported in the literature, we found in our local cohort of 125 NAFLD-associated HCC cases, of whom > 85% had histological data available for review, that of the 20% of patients who did not have underlying cirrhosis or advanced fibrosis, none were of Hispanic background. However, the remaining cohort that had NAFLD-associated HCC cases in cirrhotic livers was Hispanic (manuscript submitted). Our findings and that of others suggest that although there are genetic predispositions to NAFLD and NASH, NAFLD-associated HCC may have independent mechanisms other than those at play for NAFLD disease progression and fibrosis. Further studies that are more inclusive of patients of non-European descent are needed to determine if these associations remain true in other populations.

Beyond germ-like mutations that may predispose to disease, which has largely been the focus of genetic studies in NAFLD, heritable epigenetic changes also have an important role in NAFLD-associated HCC which include but are not limited to DNA methylation, chromosomal looping interactions, RNA modifications and the emerging role of non-coding RNAs^[54–57]^. Additionally, several studies have applied circulating tumor DNA (cfDNA) methods to further study HCC^[58,59]^, which has not only shed some light on the biology of HCC but is also promising for use as a biomarker in the future. The identification of these epigenetic modifications points to further understanding of gene regulation changes, which are influenced by their environment^[54]^.

## NON-GENETIC ENVIRONMENTAL FACTORS FOR NAFLD-HCC: CLINICAL, PHARMACOLOGICAL AND LIFESTYLE FACTORS

### Diabetes

NAFLD is a complex trait with common and rare variants^[51]^ that are influenced by the environment. Clinical studies have demonstrated that the features of MetS affect NAFLD-associated HCC development. T2D has been known to affect the risk of HCC as early as when NAFLD and NASH were starting to become more recognized. The seminal VA study by El-Serag *et al.*^[60]^ demonstrated in a cohort of 173,643 Veterans followed for 10 years that T2D significantly increased the risk of HCC. Studies that followed not only corroborated these findings^[61,62]^ but also demonstrated an increase in the risk of HCC when more features of MetS were present^[63]^. Using a prospectively collected cohort in the Nurses’ Health Study and Health Professionals’ Health Study, Simon *et al.*^[63]^ found that the adjusted hazard ratio (HR) for HCC in patients with diabetes was 5.8 (95%CI: 3.49-9.64) and 5.49 (95%CI: 3.16-9.51) compared to non-diabetic patients in women and men after adjusting for baseline characteristics, respectively. Interestingly, the risk of HCC was also dependent on the duration of diabetes, solidifying the effects of T2D and insulin resistance on hepatocellular carcinogenesis. Compared to patients without diabetes, diabetic patients had an adjusted HR of 7.52 (95%CI: 3.88-14.6) if they have had the disease for 10 years or more.

### Obesity

Obesity has been associated with an increased risk of developing many cancers and this association is strongest for HCC^[64,65]^. By convention, BMI has been used to measure obesity in epidemiological studies. Although readily available in the clinical setting, BMI does not inform adipose distribution, specifically visceral versus peripheral, which have different implications on metabolic health. Early studies in patients with cirrhosis demonstrated that those with visceral adiposity were at higher risk of death compared to those with peripheral adipose tissue. Ioannou *et al.*^[66]^ elegantly demonstrated these associations using the National Health and Nutritional Examination Survey where patients were categorized based on central or peripheral adipose distribution. Among patients with central adipose distribution, cirrhosis-related death and hospitalizations were more common in the obese group (BMI ≥ 30 kg/m^2^) (adjusted HR = 2.2, 95%CI: 1.1-4.6) compared to normal-weight individuals (BMI < 25 kg/m^2^), which was not observed in patients with increased peripheral adipose distribution. In NAFLD and NAFLD-associated HCC, central obesity, a key feature of MetS, is also more physiologically informative of metabolic health^[67,68]^. This is also relevant in the setting of studying NAFLD in groups of patients that may not have similar body compositions such as the Asian population, which tends to have a higher percentage of body fat compared to White patients^[69]^.

### Hypertension and dyslipidemia

The evidence for hypertension, which is included in many definitions of MetS, is inconsistent and has been shown to be a risk factor in some studies but not others^[70,71]^. Many studies also use collective features of MetS to assess the attendant risk. Thus, the true effects of hypertension in isolation without other features of MetS are unclear. Similarly, the data on dyslipidemia is conflicting. For instance, Welzel *et al.*^[70]^ demonstrated that patients with a diagnosis of dyslipidemia based on ICDs demonstrated an adjusted odds ratio (OR) of 1.35 (95%CI: 1.26-1.45) for NAFLD-HCC development, similar to others^[63]^. Other studies have shown the opposite effect however, in which a diagnosis of dyslipidemia was protective against HCC^[72]^. One potential reason for these conflicting reports is the definitions used to identify patients (i.e. ICDs versus lipid measurements versus medication use). These data must be interpreted in the context of statin use, which has more recently been shown to have chemoprotective effects against fibrosis progression and HCC^[14,15]^.

### Statins

Early animal studies showed that statins, which inhibit the 3-hydroxy-3-methylglutaryl coenzyme A reductase (HMG-CoA reductase), cause hepatotoxicy. The level of hepatic injury did not translate to humans clinical trials however, and mostly caused asymptomatic abnormal aminotransferases that would resolve with time^[73]^. Over the years, research and subsequently clinical practice has changed to favor the use of statins in NAFLD and NASH patients for the cardiovascular protective effects, which are closely linked to NAFLD^[74,75]^. Independent of its association with cardiometabolic disease, plasma lipidomic studies in NAFLD and NASH patients have also shown that lipid dysregulation is important in NAFLD pathogenesis, where levels of palmitoleic and oleic acids are increased^[76–78]^. These plasma levels were reflective of an increase in the activity of certain lipid enzymes, such as stearoyl-CoA desaturase 1 (SCD1), the rate limiting enzyme in monounsaturated fatty acids^[77,78]^, which is now being targeted in phase 3 clinical trials for the treatment of NAFLD^[76,79,80]^. Plasma (whole body) and liver lipid compositions also do not always correlate such that understanding lipid dysregulation becomes even more complex^[78]^.

Over the years studies have showed the benefits of statins for patients with cirrhosis. In a retrospective cohort analysis conducted within the VA from 2008-2016 that included 21,921 patients on statins and 51,023 controls without statins, Kaplan *et al.*^[14]^ demonstrated that for every year of statin exposure, the associated adjusted HR was 0.92 (95%CI: 0.89-0.94) for mortality. This was seen in all etiologies of cirrhosis including NAFLD and NASH, which comprised 23% of the cohort. These benefits of statins have also been reported in HCC. In another retrospective cohort within the VA from 2002-2016, Thrift *et al.*^[15]^ reported that after a diagnosis of HCC, statin users had a decreased risk of cancer-specific death with an adjusted HR of 0.85 (95%CI: 0.77-0.93). Their sub-group analysis of NAFLD-associated HCCs did not demonstrate a decrease in cancer-specific (adjusted HR = 1.80, 95%CI: 0.59–1.08) or all-cause (adjusted HR = 0.90, 95%CI: 0.73-1.10) mortalities. The benefits were evident in the group of patients with cirrhosis however, for both cancer-specific (adjusted HR = 0.80, 95%CI: 0.68-0.94) and all-cause (adjusted HR = 0.88, 95%CI: 0.79-0.98) mortalities, respectively, suggesting a fibrosis or cirrhosis-specific effect^[15]^. A nested case-control study conducted from 2002-2013 in the Republic of Korea also demonstrated a benefit from statin use with a reduced risk of HCC development (adjusted OR = 0.44, 95%CI: 0.33-0.58)^[81]^. Several meta-analyses have also confirmed these findings across different study populations, health care systems and etiologies of HCC^[82–84]^. The anti-fibrotic and chemoprotective effects of statins are thought to be potentially independent of their lipid-lowering mechanisms and through other pleiotropic effects mediated by the mevalonate pathway^[85,86]^. Among the statins, those that are lipophilic (atorvastatin and simvastatin) have been linked to reduced HCC incidence and mortality outcomes compared to hydrophilic statins (pravastatin and rosuvastatin) in patients with chronic hepatitis B or C-related HCC^[17]^. To the best of our knowledge, the differentiating effects of the type of statin used in NAFLD and NAFLD-associated HCC have not been studied.

### Aspirin

Chronic inflammation is a known risk factor for fibrosis. Non-steroidal anti-inflammatory drugs, including aspirin, have been associated with having chemoprotective effects in other malignancies including colorectal, breast, prostate and other gastrointestinal cancers^[87,88]^. Early *in vitro* studies demonstrated that aspirin had chemoprotective effects against HCC^[89]^. Sahasrabuddhe *et al.*^[90]^ assessed the association between aspirin use in a prospectively collected data of 300,504 patients in the National Institutes of Health-AARP Diet and Health Study cohort, where patients had used aspirin over the previous 12 months (73% used aspirin). Of the 250 patients who developed HCC, those on aspirin had decreased risk for both HCC (adjusted relative risk = 0.49, 95%CI: 0.39-0.61) and chronic liver disease mortality (adjusted relative risk = 0.55, 95%CI: 0.45-0.67)^[91]^. In an observational study using the Liver Cancer Pooling Project which consisted of US cohorts from the National Cancer Institute Cohort Consortium, of the 679 patients who developed HCC, those who were on aspirin also had a 32% reduction in the risk of HCC (adjusted HR = 0.68, 95%CI: 0.57-0.81) over the followup period of 11.9 years^[92]^. Although there was no mention of NAFLD patients (mostly hepatitis B and C), this early study demonstrated a potential benefit of aspirin in other cirrhosis populations at risk for HCC. These data led to a more recent study using the Nurses’ Health Study and the Health Professionals Follow-up Study, where over a median follow-up of 8 years, 133,371 patients were assessed for HCC development. Regular users of aspirin (≥ 2 doses; 325 mg per week) had a reduced risk of HCC (adjusted HR = 0.51, 95%CI: 0.34-0.77), which appeared to be dose and time-dependent^[16]^. Patients who used aspirin for 5 years or more had the lowest risk of HCC development (adjusted HR = 0.41, 95%CI: 0.21-0.77). Again, the etiology of HCC and cirrhosis was not evident in the study, although one would expect these data to extrapolate to NAFLD-associated HCC^[16]^. This is further supported by NASH animal models treated with aspirin demonstrating a decrease in hepatic fibrosis through decreased activation of pro-inflammatory pathways, potentially by altering the microbiome^[93]^. Further studies will be needed to ascertain this however, especially given the benefit of aspirin in cardiovascular disease, which is one of the leading causes of mortality in that patient population^[94]^.

### Microbiome

Advancements in technology, accessibility to sequencing and manipulation of big data has introduced tools to start understanding how the microbiome contributes to NAFLD, NASH and HCC progression. There is increasing evidence that gut dysbiosis plays a key role in driving the progression of NAFLD to NASH and liver cirrhosis by creating a micro-environment which supports: (1) altered energy absorption^[95]^, (2) modification of gut permeability^[96,97]^, (3) promotion of chronic low-level inflammation[^98]^ and, (4) dysregulation of bile acid signaling^[96,99–101]^. There is however, a limited understanding of the unique relationship between intestinal microbiota, dysbiosis and the development of HCC.

Studies have consistently shown that two phyla of bacteria are dominant within the gut flora, Firmicutes and Bacteroidetes and that their ratios are altered in NAFLD/NASH patients when compared to healthy controls^[100,102–105]^. In a recent study using whole-genome shotgun sequencing of DNA extracted from stool samples, Loomba *et al.*^[106]^ analyzed the microbiota of subjects with worsening degrees of fibrosis. The authors discovered that in patients with mild/moderate (F0-F1) fibrosis, the gut flora is dominated by Firmicutes and Bacterioidetes followed by Proteobacteria and Actinobacteria. In contrast, Proteobacteria levels were augmented in patients with severe fibrosis (F ≥ 2) while levels of Firmicutes were diminished. In patients with advanced fibrosis, *Bacteroides vulgatus* and *Escherichia coli* (*E.coli*) were the most abundant organisms. Given that advanced fibrosis is a risk factor for HCC, it is conceivable that similar differences may be found in patients with HCC. Indeed, a study performed by Grat *et al.*^[107]^ comparing the stool composition of patients with and without HCC (matched by etiology of cirrhosis and MELD scores) discovered that patients with HCC had significantly higher levels of *E. coli* in their stools.

Studies have also shown that alcohol espouses gut permeability through the alteration of tight junctions in gut epithelium^[108]^. With the liver being a first-pass organ, it is likely that subjects with gut dysbiosis are predisposed to inappropriate translocation of gut bacteria and their endotoxins, leading to a chronic state of inflammation. This is supported by data from Ponziani *et al.*^[109]^ who showed that compared to patients with NAFLD cirrhosis without HCC, those with HCC have higher levels of fecal calprotectin - a surrogate measure of gastrointestinal inflammation. Furthermore, Ponziani *et al.*^[109]^ demonstrated that independent of HCC, patients with compensated liver cirrhosis have higher levels of plasma lipopolysaccharide (LPS). LPS is a well-known endotoxin that simulates toll-like (TLR) and nod-like receptors in the intestinal epithelium. NASH patients have been shown to have higher expression of circulating LPS levels as well as localization to hepatocytes, which subsequently leads to activation of TLR4 and pro-fibrotic pathways^[93]^. Overexpression of TLRs leads to overproduction of chemokine ligand (CCL) 3, CCL4, CCL5 and interleukin (IL) 4, proinflammatory proteins known to be elevated in the presence of HCC, with IL-8 known specifically to be a hepatocarcinogen^[109]^.

Beyond modification of intestinal microbiota and overstimulation of the host’s innate immune system, dysbiosis has also been shown to disrupt bile acid regulation, where under physiological conditions, the microbiome metabolizes primary bile acids to secondary bile acids that are recirculated through the enterohepatic circulation^[110]^. When comparing patients with NAFLD to healthy controls and those with simple steatosis, Mouzaki *et al.*[^100]^ discovered that NAFLD patients had higher fecal levels of primary bile acids, specifically colic acid, chenodeoxycholic acid and lithocholic acid. Others have also shown, using animal models, that elevated levels of lithocholic acid may be carcinogenic^[100,102]^. Bile acid dysbiosis in patients with cirrhosis is also well illustrated by Jacobs *et al.*^[111]^ using duodenal aspirate analyses. Although NASH only comprised 13% of the cohort of patients with cirrhosis, the study identified microbial differences based on the etiology of the cirrhosis, cirrhosis complications (specifically patients with hepatic encephalopathy) and ethnic differences where Hispanics were found to have lower levels of two conjugated forms of ursodeoycholic acid. This is especially interesting given the ethnic differences previously described in Hispanics and the observation that ursodeoycholic acid may have anti-carcinogenic effects on HCC^[112–114]^.

The role of gut dysbiosis in creating a pro-carcinogenic environment is further supported by animal studies. Specifically, in mice models, researchers have shown that the administration of antibiotics/probiotics disrupts the development of new HCC lesions. The replacement of the pro-inflammatory gut milieu, either with sterilization or bacterial replacement, highlights once again the role of the gut-hepatic axis in HCC development, and brings to light the potential role of therapeutics in patients at high risk of HCC^[115–117]^.

Overall, gut dysbiosis has been repeatedly shown to promote metabolic diseases and accelerate the progression of fatty liver disease. There is now increasing evidence however, that the gut microbiome acts as an independent risk factor for HCC development. By promoting an environment that espouses intestinal permeability, hepatic inflammation and bile acid dysregulation, the gut microbiome creates a procarcinogenic environment that may allow for the development of HCC, potentially by working as an epigenetic regulator of gene expression. Most studies in the literature are currently limited to animal models, and of those involving human subjects, samples sizes are often limited by their understandably strict inclusion criteria. Thus, further studies are needed to elucidate the intricate relationship between gut dysbiosis and HCC. Although far from clinical application, this also opens up new avenues for biomarker discovery and potentially, therapies.

## CIRRHOSIS *VS.* NON-CIRRHOSIS

As highlighted above, work remains to be done to further understand the prevalence, causes and outcomes of NAFLD-associated HCC given its complexities with many environmental contributors. Complicating the presentation of NAFLD-associated HCC is that it can occur in a non-cirrhosis background, which causes a clinical dilemma to providers given the lack of current screening guidelines for this patient population. It is estimated that the prevalence of NASH-associated HCC in patients without cirrhosis to be 38% based on a recent meta-analysis^[11]^, which differs based on the population, country and how the study was conducted. In Japan, where patients can also have “lean” NAFLD, studies have reported that NASH-related HCC can occur without cirrhosis in 38%-49% of cases^[118–120]^. A multi-center prospective study conducted in Spain reported a prevalence of 50% of HCC in NAFLD patients without cirrhosis^[12]^. Similarly, a retrospective study in France assessing the prevalence of non-cirrhosis HCC in a cohort of 323 HCCs over a 20-year span (of which 12% were due to NAFLD) determined that 63% of the cases occurred in the absence of bridging fibrosis/cirrhosis, although this was biased towards patients without advanced liver disease who could undergo hepatic resection instead of liver transplantation^[121]^.

Interestingly, statin use or dyslipidemia also appears to have different effects on non-cirrhosis HCC patients. For instance, using Taiwan’s National Health Insurance Research Database that included 31,751 NAFLD patients from 1998-2012, Lee *et al.*^[13]^ demonstrated that patients on statin therapy (35% of all patients) had a decreased risk for HCC (adjusted HR = 0.29, 95%CI: 0.21-0.68). Few other studies have addressed the effects of statins between the cirrhosis and non-cirrhosis NAFLD patient, which necessitates future work in this area.

**Figure 1.**
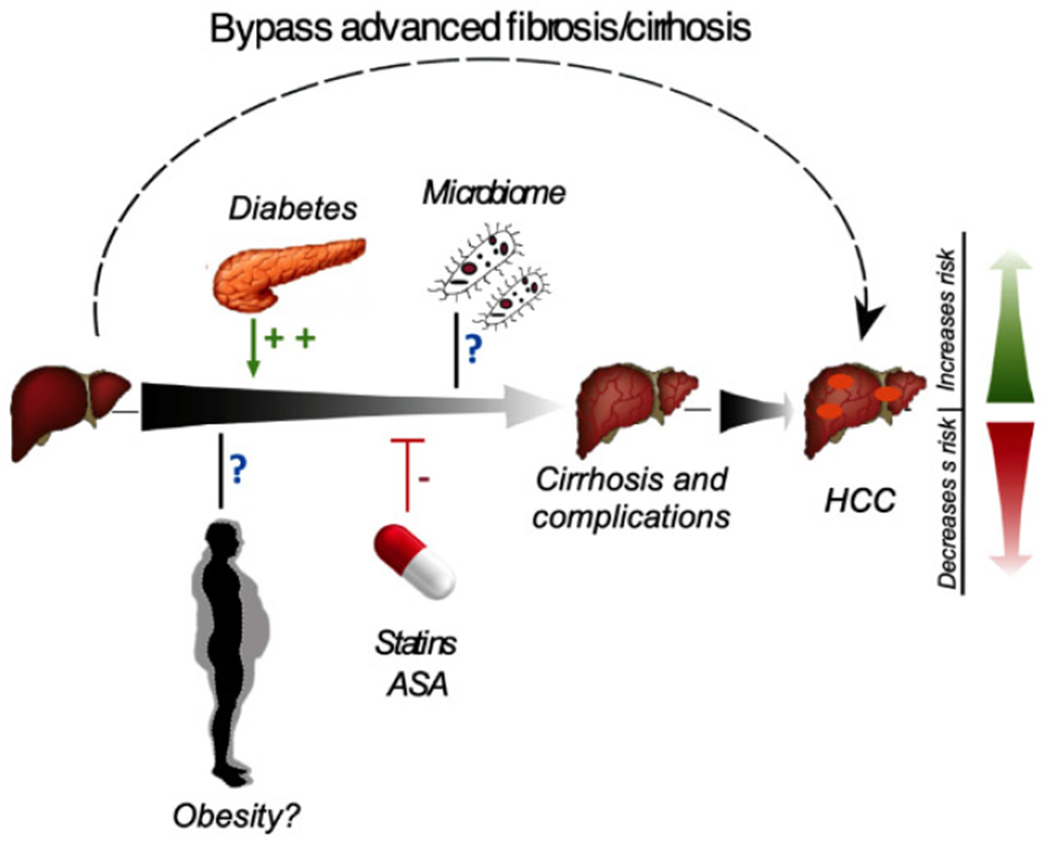
Schematic diagram of the spectrum of NAFLD to NAFLD-associated HCC and the clinical factors implicated in its pathogenesis or prevention. NAFLD: nonalcoholic fatty liver disease; HCC: hepatocellular carcinoma; ASA: aspirin

Animal studies have shed some light on the pathogenesis of NAFLD and NAFLD-associated HCC. Through a series of detailed experiments, Grohmann *et al.*^[122]^ demonstrate that while obesity promotes NASH pathogenesis through STAT-1 (signal transducer and activator of transcription 1) activation, NASH-associated HCC is mostly driven by STAT-3 (signal transducer and activator of transcription 3), a transcription factor implicated in the immune system. They further corroborated this using human samples as proof of concept. Their work was complimentary to previous reports showing that STAT-3 activation correlates with tumor aggressiveness and prognosis^[123,124]^. As our knowledge of NAFLD and NAFLD-associated HCC advances, it is becoming evident that the two diseases are divergent and should, potentially, be thought of as separate entities and not on the same spectrum [Figure 1]. This is also apparent in our epidemiological studies where we found that NAFLD-associated HCC tended to occur in non-Hispanic patients, suggesting different mechanisms of action between the cirrhosis and non-cirrhosis patient populations (manuscript submitted).

## SCREENING?

Expert societies recommend HCC screening with bi-annual ultrasounds with or without alpha-fetal protein (AFP) or other biomarkers, based on its cost-effectiveness and benefits^[125,126]^. Although the incidence of NAFLD-associated HCC remains low overall, given the magnitude of MetS and the NAFLD epidemic, NAFLD-HCC cases are expected to increase. Since HCC can also occur in a non-cirrhotic background, future research is imperative in this field to identify at-risk patient populations that would benefit from screening programs, which have largely operated under a “one-size-fits-all” model. Unlike hepatitis C, B and alcohol, NAFLD HCC screening has several challenges. Obesity, common in this patient population, has been shown to decrease the effectiveness of ultrasound screening^[127–129]^. The effects of visceral adipose tissue or intrahepatic steatosis in lesions are also unclear. The use of other imaging modalities has been proposed including abbreviated MRI scans, which are promising given their sensitivity and specificity, although their cost-effectiveness remains to be evaluated^[130,131]^.

Over the years, new biomarkers have been introduced including the Lens culinaris lectin-binding subfraction of the AFP (AFP-L 3%)^[132]^ and des gamma carboxyy prothrombin^[133]^. The clinical utility of these biomarkers in NAFLD-associated HCC patients remains unknown. In our studies, we found that compared to hepatitis B and C, patients with NAFLD-associated HCC were less likely to be AFP producers (AFP < 10 ng/mL) (manuscript submitted). Due to the complex nature of NAFLD, the use of a combination of markers will likely be more clinically relevant. This is demonstrated by Best *et al.*’s^[134]^ study using the GALAD (Gender, Age, AFP-L3, and Des-carboxy-prothormbin) score to predict early detection of NAFLD-associated HCC cases in Europe and Japan, including the sub-group of patients without cirrhosis. These results will need to be validated in the US patient population given the heterogeneity of NAFLD across different ethnic groups, especially given the lower performance of the GALAD score in the sub-group of US-American cohort. Risk stratification calculators have been developed to assess the risk of HCC and the utility of HCC screening, including in the NAFLD patient population, however these have focused on the cirrhosis group which is at higher risk of HCC and have not entered clinical practice yet^[24]^.

Clinical practices therefore vary due to the limited data, which has prompted expert societies to provide some guidance in the form of expert opinion^[135]^. In our local liver transplantation institution, we choose to screen patients yearly with an abdominal ultrasound and blood work, especially if they have a diagnosis of T2D.

## CONCLUSIONS AND OUTSTANDING QUESTIONS

NAFLD-associated HCC is on the rise and will continue to create a large economic burden on health care, prompting essential research. Its heterogenous clinical presentation is reflective of its complex traits with important genetic and non-genetic, as well as environmental risk factors. Although a combination of basic, translational and clinical research has shed some light on its distinct presentations, many questions remain unanswered. The lack of consistent clinical definitions to identify NAFLD and NASH patients when using electronic medical records in research over the years has made comparison between studies challenging. This has been recognized in clinical trials where a larger effort has been made on using consistent and reproducible definitions to identify patients and characterize responses to NASH treatment. Bigger public health questions remain unanswered including who to screen and how to use modifiable factors such as statins and aspirin to mitigate the risk of liver disease progression and HCC, where liver transplantation remains scarce and not available to all patients. In the era of precision healthcare and medicine, identifying “at-risk” populations within the larger NAFLD group will be key to tailoring screening and treatment options and will help providers identify patients in need of close monitoring under the care of subspecialists versus primary care. In light of the complex and heterogenous nature of the disease, identifying “at-risk” patients will likely require a combination of clinical characteristics, biomarker discovery and risk-stratification calculators.

## Figures and Tables

**Table 1.** NAFLD-associated HCC epidemiology and burden

Country	Incidence and prevalence	Population	Study period	Ref.
United States	0.21 per 1000 person-years	Veterans Affairs	2003-2011	Kanwal *et al.*^[22]^
	14.1% of all cases	SEER registries	2004-2009	Younossi *et al.*^[136]^
	8% of all HCC cases	Veterans Affairs	2005-2011	Mittal *et al.*^[23]^
	1.56% of HCC cases	Veterans Affairs	2012-2018	Ioannou *et al.*^[24]^
	5.29 per 1000 person-years	Meta-analysis	1989-2015	Younossi *et al.*^[3]^
	13.5% of all liver transplants	United Network Organ for Sharing	2000-2012	Wong *et al.*^[26]^
Spain, Italy, the Netherlands, United Kingdom	0.3 per 1000 person-years	European primary care databases	2016	Alexander *et al.*^[30]^
United Kingdom	35% of all HCC referrals	National Health Services	2010	Dyson *et al.*^[137]^
Japan	6% incidence	Single hospital in Tokyo	1994-2007	Arase *et al.*^[138]^
South Korea	12.2% incidence	South Korean hospital	2006-2010	Cho *et al.*^[34]^

NAFLD: nonalcoholic fatty liver disease; HCC: hepatocellular carcinoma; SEER: surveillance, epidemiology and end results

**Table 2.** Summary of NAFLD HCC polymorphisms

SNP	Clinical significance	Location (human chromosome)	Associated gene	Role of the gene	Ref.
rs738409 C>G	Associated with increased liver fat accumulation and a higher risk for developing liver cirrhosis. Given the increased severity of necroinflammation in GG sequence carriers, these individuals may be at higher risk for developing HCC	chr22:43928847 (GRCh38.p12)	*PNPLA3*; Patatin-like phospholipase domain-containing protein 3	The*PNPLA3* gene encodes the protein Adiponutrin, which is thought to help regulate the development of adipocytes as well as lipogenesis and lipolysis in the liver	Sookoian *et al.*^[44]^ Shen *et al.*^[139]^
rs58542926 C>T	Associated with an increased risk of developing diabetes and NAFLD. The polymorphism results in a loss of function and individuals with the CT phenotype have a reduced hepatic capability to secrete LDLs and are at higher risk for developing liver inflammation and potentially HCC	chr19:19268740 (GRCh38.p12)	*TM6SF2*; Transmembrane 6 Superfamily Member 2	The *TM6SF2* gene is involved in regulating liver fat metabolism, lipoprotein secretion and hepatic lipid droplet content	Kozlitina *et al.*^[46]^ Falleti *et al.*^[47]^ Vespasiani-Gentilucci *et al.*^[140]^
rs641738 C>T	Associated with an increased risk of hepatic fat accumulation, fibrosis, and potentially HCC	chr19:54173068 (GRCh38.p12)	*MBOAT7*; Membrane Bound O-Acyltransferase Domain Containing?	The *MBOAT7* gene encodes a protein known as Lysophospholipid acyltransferase 7 that is involved in the re-acylation of phospholipids as part of the phospholipid remodeling pathway	Mancina *et al.*^[48]^ Luukkonen *et al.*^[49]^ Donati *et al.*^[50]^

NAFLD: nonalcoholic fatty liver disease; HCC: hepatocellular carcinoma; LDLs: low density lipoproteins
